# Combinatorial targeting of multiple myeloma by complementing T cell engaging antibody fragments

**DOI:** 10.1038/s42003-020-01558-0

**Published:** 2021-01-08

**Authors:** Maria Geis, Boris Nowotny, Marc-Dominic Bohn, Dina Kouhestani, Hermann Einsele, Thomas Bumm, Gernot Stuhler

**Affiliations:** Department of Internal Medicine II, Hematology and Oncology, University Clinic Würzburg, Würzburg, Germany

**Keywords:** Myeloma, Immunotherapy

## Abstract

Bispecific T cell engaging antibodies (BiTEs) address tumor associated antigens that are over-expressed on cancer but that can also be found on healthy tissues, causing substantial on-target/off-tumor toxicities. To overcome this hurdle, we recently introduced hemibodies, a pair of complementary antibody fragments that redirect T cells against cancer-defining antigen combinations. Here we show that hemibodies addressing CD38 and SLAMF7 recruit T cells for the exquisite elimination of dual antigen positive multiple myeloma cells while leaving single antigen positive bystanders unharmed. Moreover, CD38 and SLAMF7 targeting BiTEs, but not hemibodies induce massive cytokine release and T cell fratricide reactions, a major drawback of T cell recruiting strategies. Together, we provide evidence in vitro and in vivo that hemibodies can be developed for the effective and highly specific immunotherapy of multiple myeloma.

## Abstract (web summary)

Geis et al. investigate the potential application of hemibodies, a pair of complementary antibody fragments that redirect T cells against cancer-defining antigen combinations, such as CD38 and SLAMF7, to target multiple myeloma. Their study provides evidence that hemibodies can be developed for effective immunotherapy against multiple myeloma.

## Introduction

Multiple myeloma (MM) is the second most common hematological neoplasm and remains a rather uncurable disease until today^1–3^. In addition to standard therapy^4–7^, several novel immunotherapeutic strategies have been clinically investigated, including checkpoint inhibitors, monoclonal antibodies, antibody–drug conjugates, and T cell recruiting strategies based on chimeric antigen receptor (CAR) T cells and bispecific antibody derivatives.

CD38 and SLAMF7 (CS1/CD319) are target antigens stably expressed on virtually all MM cells at high levels. However, these antigens are also found in pneumocytes, lung endothelial cells, erythrocytes, and natural killer (NK) cells, explaining some of the many toxicities observed in immunoglobulin G (IgG)-based clinical trials. B cell maturation antigen (BCMA) is a marker exclusively expressed on B cells and B cell-derived malignancies. High initial response rates to CAR T cells or bispecific antibodies targeting BCMA have been reported for patients suffering from relapsed or refractory MM, but at the cost of severe side effects on some patients, most dominantly neurologic toxicity and cytokine release-related issues^2,8–10^. By and large, while single-agent therapy with monoclonal antibodies or antibody–drug conjugates often lack efficacy, the more potent T cell recruiting strategies induce a high level of off-tumor toxicity.

Consequently, we here study a two-component targeting approach designed to activate T cells exclusively on-target cells, thus avoiding CRSs, neurotoxicity, and innocent bystander cytolysis. Exploiting novel bi-molecular antibody derivatives coined hemibodies^11^, we demonstrate that MM regularly co-expresses CD38 and SLAMF7 antigens at high levels, which can be addressed by a combinatorial immunotherapy. Precisely, a hemibody pair addressing CD38 and SLAMF7 redirects T cells against dual antigen-positive myeloma cells in vitro and in vivo, while sparing single antigen-positive bystanders. In aggregate, the results presented here provide experimental evidence for a precise and highly potent treatment of MM.

## Results

### Co-expression of SLAMF7 and CD38 on primary MM

**Fig. 1:**
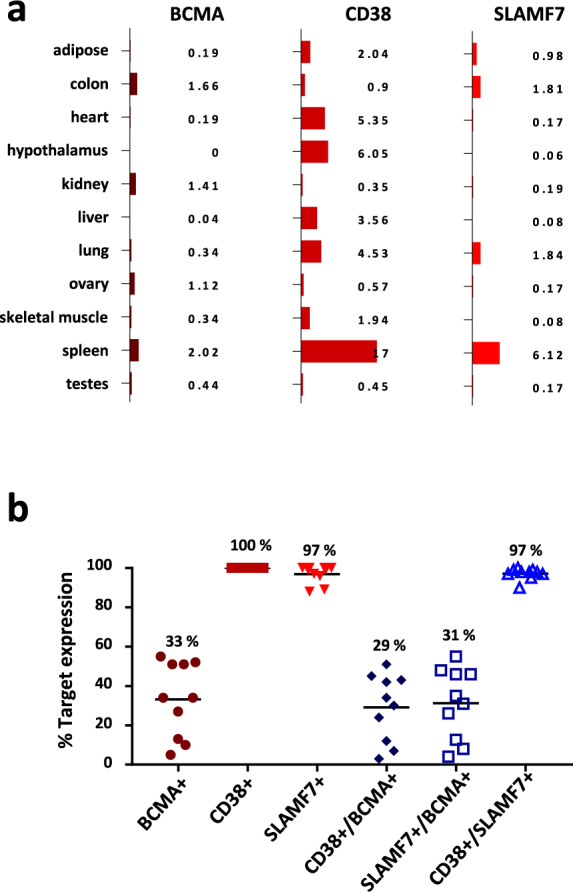
Combinatorial expression of BCMA, CD38, and SLAMF7 antigens on multiple myeloma and healthy tissues. **a** mRNA expression patterns of BCMA, CD38, and SLAMF7 on healthy tissues by publicly available Cellfinder software. **b** Co-expression of BCMA, CD38, and SLAMF7 on primary MM cells. The protein expression level of selected targets was evaluated on ten primary myeloma cell populations by flow cytometry techniques (*n* = 10, pooled data are shown).

To investigate the potential benefit of combinatorial targeting of tumor cells using the complementing hemibody technique, we resorted to an MM model system focusing on three antigens that are clinically addressed by monoclonal antibodies and T cell-redirecting strategies in ongoing trials. In the first set of experiments, we analyzed messenger RNA (mRNA) expression patterns of the BCMA, CD38, and SLAMF7 on MM cells and healthy tissues using publicly available information and Cellfinder software (Fig. 1a). In line with previously established results, we found BCMA largely restricted to the B cell compartment. In addition to the wide expression on hematopoietic cells, CD38 is detected in the heart muscle, hypothalamus, liver, and lung; SLAMF7 in colon and lung tissues, NK cells, and activated T cells.

After informed consent was obtained, we next assayed expression levels of the BCMA, CD38, and SLAMF7 antigens on 30 primary myeloma cells from clinical bone marrow samples collected at our institution. While CD38 and SLAMF7 were detected at high levels on the surface of virtually all myeloma cells even in heavily pretreated patients, we found heterogeneous BCMA expression with substantial inter- and intra-individual differences and the regular occurrence of BCMA-negative subpopulations (Fig. 1b). The importance of these data is evidenced by recent clinical trials that report a high overall response rate after BCMA-reactive BiTE or CAR T cell treatment but also the frequent occurrence of BCMA-negative relapses during the first 12 months following such T cell-redirecting therapies^10^.

### Logic AND-gate immunotargeting by hemibodies

**Fig. 2:**
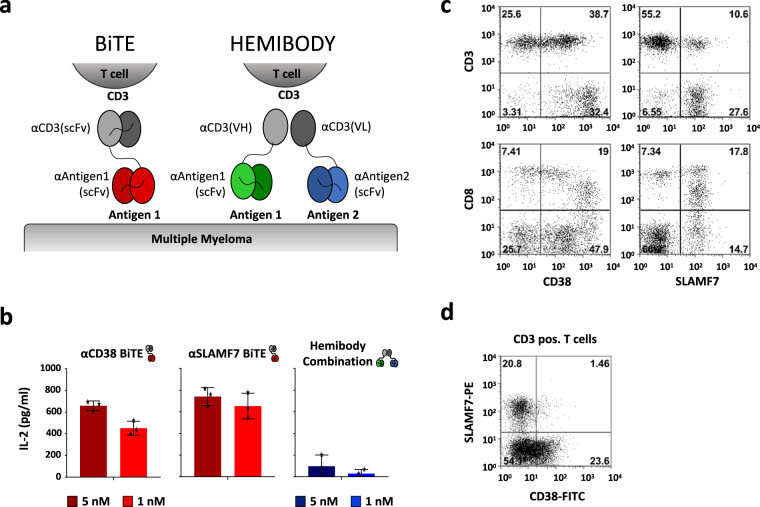
T cell engagement induced by hemibodies and BiTEs. **a** Complementation of a functional CD3-binding site and recruitment of T cells on-target by hemibodies in comparison to BiTE antibodies. Each hemibody consists of a single-chain variable fragment (scFv) fused to the variable light (Vl) or variable heavy-chain domain (Vh) of a CD3-specific antibody. Designed hemibodies address CD38 (VlαCD3-scFvαCD38; blue) or SLAMF7 (VhαCD3-scFvαSLAMF7; green). Cognate bispecific antibodies of the BiTE class (scFvαCD3-scFvαCD38/SLAMF7; red) were engineered as comparators. **b** Antibody-mediated T cell activation of 50,000 PBMCs in the absence of tumor cells was assessed by the release of interleukin-2 (IL-2) after 24 h incubation with 5 and 1 nM constructs using ELISA techniques. PBMCs were treated with 1× PBS, single hemibody constructs, and 0.5 µg T cell-activating concanavalin A served as controls (not shown). Data represent the mean value (given as *) ± SD of three individual experiments. **c** A total of 5 × 10^5^ PBMCs were analyzed for their protein expression of CD38 and SLAMF7 on CD3^+^ and CD8^+^ T cell subpopulations by flow cytometry (*n* = 3, single representative experiment is shown). **d** A total of 2 × 10^5^ CD3^+^ T cells were analyzed by flow cytometry for the co-expression of CD38 and SLAMF7 (the single experiment is shown).

In light of these observations, we set out to test a combinatorial approach employing a bi-molecular T cell engager that targets the combination of CD38 and SLAMF7. Combinatorial elimination of tumor cells is realized by a T cell engaging device, split into two complementary parts. Each fragment, coined hemibody, is composed of an antigen-binding site (a single-chain fragment variable, scFv) fused to a split T cell engaging unit, the heavy- (Vh) or light-chain variable domain (Vl) of an anti-CD3 antibody (Fig. 2a, a BiTE molecule is presented for comparison). After simultaneous binding of the hemibody pair to their targets on the surface of a single tumor cell, the Vh and Vl domains align and reconstitute the CD3-binding site for the recruitment and activation of T cells. Consequently and based on the previous evidence^11^, we engineered CD38 and SLAMF7 targeting bispecific T cell engagers (BiTEs) and hemibodies addressing the combination of the two antigens. Information on design, production, isolation, and antigen binding of the hemibody and BiTE constructs is detailed in Supplementary Figs. 1 and 2.

BiTEs and hemibodies were next tested in T cell cultures in the absence of tumor cells, because we detected the substantial expression of CD38 and SLAMF7 on CD3 and CD8 T lymphocytes, albeit distinct T cell subpopulations expressed the antigens in a mutually exclusive pattern (Fig. 2c, d). We found BiTEs addressing CD38 and SLAMF7 to induce T cell fratricide reactions and massive interleukin-2 (IL-2) secretion (Fig. 2b). These findings are highly indicative of the potential initiation of a cytokine release syndrome (CRS) by CD38- or SLAMF7-directed bispecific antibodies, a life-threatening clinical situation and a major drawback of current T cell engaging strategies. In stark contrast, low cytokine production was observed for the hemibody pair, although both molecules bind comparably well to antigen-positive cells (Supplementary Figs. 1 and 2) with a *K*_D_ of 61 and 81 nM for the CD38 and SLAMF7 targeting hemibody. We interpret this finding against the background of imperative co-expression of the antigens on the surface of a single target cell, which is not the case for the distinct single antigen-positive T cell populations as shown in Fig. 2d^11^. Hemibodies, in this respect, discriminate single versus dual antigen-positive cells on single-cell level, thus establishing a Boolean logic AND-gate device.

### Ultra-precise elimination of MM in vitro and in vivo

**Fig. 3:**
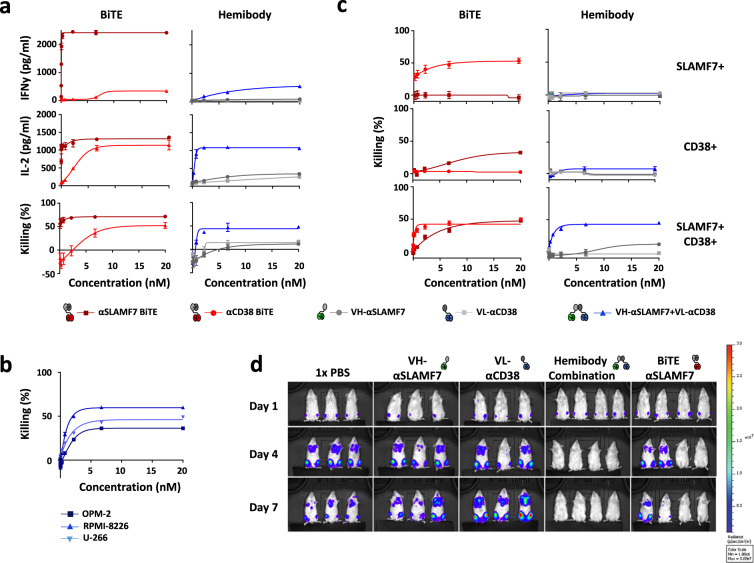
Ultra-precise immunotherapy by hemibodies in vitro and in vivo. **a** Luciferase-positive MM.1S cells were co-cultivated with PBMCs (ratio effector:target cell = 5:1) and constructs as indicated for 24 h. Antibody-initiated T cell activation was measured by IL-2 and IFN-γ secretion (top two panels) using ELISA and tumor cell lysis by luciferase-based cytotoxicity assay (bottom panel) (IL-2 and IFN-γ *n* = 3, cytotoxicity *n* > 3, pooled data are shown). **b** Luciferase-positive MM cell lines OPM-2, RPMI-8226, and U-266 were co-cultivated with PBMCs and different construct concentrations for 24 h. Data represent the mean values with standard deviations (±SD) from three independent experiments. The effector to target cell ratio was 5:1. Tumor cell lysis was determined by the intracellular luciferase activity of living cells. **c** Luciferase-positive CHO cells expressing either CD38 or SLAMF7 (top two panels) or both antigens (bottom panel) were co-cultivated with PBMCs (effector:target cell ratio = 5:1) and constructs as indicated. BiTE- and hemibody-mediated tumor cell lysis was assessed using luciferase-based cytotoxicity assay. Cognate BiTE constructs are shown for comparison (*n* = 3, pooled data are shown). **d** For in vivo analysis of hemibodies, 2 × 10^6^ luciferase-positive MM.1S cells were injected intravenously (i.v.) in immunodeficient NOD SCID mice. After tumor cell engraftment (day 0 of treatment) mice were treated once with 1 × 10^7^ PBMCs, followed by daily subcutaneous applications of either 1× PBS, hemibodies addressing SLAMF7 or CD38, the combination of both hemibodies, or SLAMF7-specific BiTE controls (8 µg/mouse per day for one week) (*n* = 2, a single experiment is shown). Tumor growth of luciferase-positive MM.1S cells was detected by IVIS Lumina XR Real-Time Bioluminescence Imaging.

We next analyzed the capabilities of hemibodies to recruit T cells for lysis of myeloma cells and to secrete cytokines. Single hemibodies specific for either CD38 or SLAMF7 exerted only minimal T cell recruiting capability when co-cultured with the dual antigen-positive MM.1S myeloma cell line, even at very high concentrations of the constructs (Fig. 3a). The combination of hemibodies, however, induced T cells to secrete IL-2 and interferon-γ (IFN-γ) and to efficiently lyse myeloma cells (right panel) to almost the same extent as BiTE molecules, which are given as comparators (left panel). Together, these results indicate high activity of the hemibody constructs after complementation and reconstitution of the CD3-binding site after binding of both hemibodies to myeloma cells. This effect of hemibody-induced myeloma cell lysis was validated and verified on three further myeloma cell lines (OPM-2, RPMI-8226, and U-266; Fig. 3b).

Having obtained this information, we next set out to test the capabilities of hemibodies to discriminate dual antigen-positive target cells from single positive bystanders. To this end, we established Chinese hamster ovary (CHO) cell lines expressing CD38, SLAMF7, or the combination of the two antigens and investigated BiTEs and hemibodies to engage T cells against the respective targets. As shown in Fig. 3c, dual antigen-positive CHO cells (bottom panel), but not single positives (top two panels) were lysed by T cells after incubation with the cognate hemibody combinations (blue curves). Individual constructs only marginally stimulated T cell cytotoxicity. The results for BiTEs are presented as comparators. As expected, BiTEs redirect T cells to targets expressing the cognate antigen without discriminating dual versus single antigen positivity. Together, these findings indicate that hemibodies, but not BiTEs, redirect T cells with unprecedented precision exclusively towards dual-positive targets. Single antigen-positive cells representing healthy bystander cells and tissues are spared.

Based on the favorable co-expression of CD38 and SLAMF7 antigen at high levels on MM, we tested hemibodies in vivo using myeloma xenografts and human peripheral blood mononuclear cells (PBMCs) in a humanized immunodeficient NOD SCID *Il2rg*^−/−^ (NSG) mouse model (Fig. 3d). After engraftment of human luciferase-positive MM.1S cells 14 days after intravenous (i.v.) injection, 10E7 human PBMCs were given i.v., followed by subcutaneous administration of 8 µg of the constructs per day as indicated. As shown in Fig. 3d, tumors grew in mice receiving saline or individual hemibodies. In contrast, mice receiving the hemibody combination or BiTEs rejected established tumors within the first 4 days of treatment.

## Discussion

Despite the plethora of innovative drugs that enter the clinical stage to address the complex and highly heterogeneous genetic landscape of MM, remissions are short and cures are rare. Although promising data from phase I/II trials have been reported for T cell recruiting immune therapeutic strategies, no long-term disease control without significant toxicity has been observed in patients with a more advanced disease.

The reasons for treatment failure are twofold and are based on the selected antigen: first, some target antigens are heterogeneously expressed within the myeloma cell population with individual clones displaying downregulated antigen levels or even antigen loss. This point is imminent in current clinical studies for BCMA-directed immunotherapies where relapses after initial high response rates are being observed and reported. In contrast to BCMA, CD38 and SLAMF7 antigens show stable expression levels throughout the successive lines of MM treatment. Second, the target antigen is expressed on tumor cells and healthy tissues alike. This situation is reported for CD38 and SLAMF7 antigens. Addressing these molecules using antibodies or antibody–drug conjugates lead to dose-limiting on-target/off-tumor toxicities. These innocent bystander effects, however, may become severe, if not lethal, when high-performing T cell engaging immunotherapies like CAR T cells or bispecific antibodies are employed.

The expression of CD38 and SLAMF7 on T lymphocytes is widely appreciated and immunotherapies addressing these molecules interfere with T cell survival and function. For CD38-specific IgG, immunosuppressive effects are readily observed including reactivation of hepatitis B, most likely because of a severe impairment of the T cellular compartment. Moreover and as shown here, redirecting T cells against CD38 or SLAMF7 antigens that are expressed on distinct T cell populations leads to a substantial release of immunostimulatory cytokines as part of a fratricidal reaction, accompanying impairment of the therapeutic response. Clinically, cytokine release syndromes and associated neurotoxic side effects are major drawbacks of T cell recruiting strategies, limiting therapeutic efforts and successes.

In light of these drawbacks, we here investigated a combinatorial approach using a complementary pair of hemibodies that target the aberrant antigen combination of CD38 and SLAMF7 expressed on MM. We found exquisite lysis of dual antigen-positive myeloma cells by this hemibody pair while single antigen-positive bystander cells are spared. This beneficial effect of combinatorial specificity has been observed for the hemibody pair but not for the BiTE comparators and may translate into very low off-tumor toxicity in clinical settings.

Importantly, antigens addressed by hemibodies do not need to be tumor specific. Any antigen can be used as long as the combination with the partner is uniquely expressed on the target population. As reported by protein expression, databases like the human protein atlas SLAMF7 and CD38 are not co-expressed on healthy tissue. Consequently, even widely expressed antigens like CD38 or SLAMF7 can be addressed by this split antibody approach.

## Methods

### Cell lines

The CD38- and SLAMF7-positive MM cell lines OPM-2, RPMI-2668, U-266, and MM.1S were purchased at the German Collection of Microorganisms and Cell Cultures (DSMZ, Braunschweig, Germany; DSMZ nos.: ACC50, ACC402, ACC9, and ACC758) and engineered to express the *firefly luciferase* (*ffluc*) gene, kindly provided by Michael Hudecek (University Hospital Wuerzburg, Wuerzburg, Germany). All cell lines were cultivated in advanced RPMI-1640 supplemented with 200 μM l-glutamine, 10% fetal bovine serum (FBS), penicillin (200 U/ml), and streptomycin (200 μg/ml) (Thermo Fisher Scientific, MA, USA).

CHO (DSMZ, Braunschweig, Germany, DSMZ no.: ACC 110) were transduced with the *ffluc* and full-length *CD38* and/or *SLAMF7* gene using the PiggyBac cDNA Cloning and expression Vector PB-CMV-MCS-EF1α-Fluc (PB514B-2, System Biosciences, LLC, Palo Alto, CA, USA). A total of 0.3 × 10^6^ adherent CHO cells in 2 ml F-12K media were transfected by 250 μl DNA–lipid complex consisting of 12.5 μl Lipofectamine^TM^ 2000 Transfection Reagent (Thermo Fisher Scientific, MA, USA), 1 μg PB transposase, and 1.5 μg of PB vectors in Opti-MEM media for 48 h at 37 °C. Single or dual antigen-positive CHO cells were selected by the expressed antigen levels by fluorescence-activated cell sorting (FACS). The sequences used for CD38 and SLAMF7 were obtained from the National Center for Biotechnology Information website (www.ncbi.nlm.nih). The cells were cultivated in F12-K medium supplemented with 200 μM l-glutamine, 10% FBS, penicillin (200 U/ml), and streptomycin (200 μg/ml) (Thermo Fisher Scientific, MA, USA). All cell lines were tested negative for mycoplasma. Authentication of the cells was performed by the provider.

### Human PBMC/T cell Isolation

PBMCs were purchased from the University Hospital Würzburg in agreement with institutional consent and collection guidelines (Institute for Transfusion Medicine and Haemotherapy, University Hospital Wurzburg and Wurzburg University, Ethic Committee Approval No. 141/17-sc) and isolated by density gradient centrifugation. T lymphocytes were further purified using the Pan T Cell Isolation Kit (Miltenyi Biotech) according to the manufacturer’s guidelines. PBMCs/T cells were maintained until usage in advanced RPMI-1640 media supplemented with FBS (10% (v/v)), l-glutamine (1% (v/v)) and penicillin/streptomycin/neomycin (1% (v/v)) at 37 °C and 5% CO_2_.

### Hemibody construction

DNA coding for hemibodies and bispecific BiTEs were synthesized by GeneArt™ (Gene Synthesis, Thermo Fisher Scientific Inc., Regensburg, Germany) using published scFv sequences of antibodies targeting CD38 (Morphosys Ag, US 20160115243 A1, “Anti-cd38 human antibodies and uses thereof”, 6. Febr. 2004), SLAMF7 (sequence of Elotuzumab, Drug Bank DB06317), and CD3ε (diL2K, the de-immunized version of the mouse monoclonal antibody L2K, Micromet/Amgen)^12^. In addition, a N-terminal fused Trx1 tag was used for an enhanced cytoplasmic protein expression, combined with a C-terminal 8× histidine tag for purification (Supplementary Fig. 1a). For antibody production, hemibodies were cloned into a pCOLDIV plasmid (TaKaRa Bio Inc., Japan; Supplementary Fig. 1a).

### Prokaryotic protein expression

Hemibodies and BiTE constructs were expressed in Shuffle T7 *Escherichia coli* cells (New England Biolabs, MA, USA, NEB3029) in 2× YT medium supplied with 0.1% v/v glucose, 75 μg/ml carbenicillin, and 37.5 μg/ml kanamycin within 20 h at 14 °C and induced by the addition of 1 mM isopropyl-β-d-thiogalactopyranosid. Solubility tags were removed by the cleavage of a co-expressed 3C protease on a pRSF Duet plasmid. Afterwards, bacterial cells were lysed and hemibodies purified by affinity chromatography at 4 °C.

### Immobilized metal affinity chromatography (IMAC), desalting, ion exchange, and size-exclusion chromatography (SEC)

Hemibodies were purified by IMAC via 8× histidine tag by loading the *E. coli* lysate onto a 5 ml HiTrapTALON crude column (GE Healthcare Bio-Sciences, PA, USA) at 5 ml/min using the ÄKTA Start Chromatography System (GE Healthcare Bio-Sciences, PA, USA). Unbound proteins and endotoxins were removed by washing with 5 column volumes (CV) wash buffer (50 mM Na phosphate pH 7.5, 300 mM NaCl, 10 mM imidazole pH 8.0), 50 CV endotoxin removal buffer (50 mM Na phosphate pH 7.5, 300 mM NaCl, 5 mM imidazole pH 8.0, 0.2% Triton X-114) and 10 CV wash buffer. Bound hemibodies and BiTEs were eluted with 5 CV elution buffer. Eluted antibodies were desalted using HiPrep 26/10 desalting column (GE Healthcare Bio-Sciences, PA, USA) for buffer exchange to anion exchange buffer (AIEX, 50 mM Na phosphate pH 7.5, 75 mM NaCl) and further purification steps by anion exchange chromatography on a 1 ml HiTrap Q FF column (GE Healthcare Bio-Sciences, PA, USA) at 1 ml/min. Afterwards, constructs were subsequently purified by SEC on a HiLoad 16/600 Superdex 200 pg column (GE Healthcare Bio-Sciences, PA, USA). Constructs were loaded, separated, and eluted, according to their size, in SEC buffer (50 mM Na phosphate pH 7.5, 300 mM NaCl) at 1 ml/min. Desalting/buffer exchange, AIEX, and SEC purification steps were performed at RT using ÄKTA Pure Chromatography System (GE Healthcare Bio-Sciences, PA, USA). An overview and analysis of the hemibody production and purity after IMAC, delalting/buffer exchange, IEX, and SEC is given in the Supplementary Fig. 1b.

### Flow cytometry

Flow cytometry was performed by standard methods using a FACSCalibur device (BD Biosciences, Heidelberg, Germany). Data files were analyzed using the FlowJo software version 8.8.7 (Tree Star Inc., Ashland, USA). For FACS analysis on primary MM samples, informed consent was obtained from all patients and sample collection and handling was conducted in accordance with the provisions of the Declaration of Helsinki.

### Antigen expression

Selected tumor antigens were detected on 2 × 10^5^ T cells or primary MM cells using 0.25 µg fluorochrome-labeled FACS antibodies (α-BCMA Clone Vicky-1 Novus Biologicals/Clone 19F2, BioLegend, CD38 Clone HB-7 BioLegend/Clone LS198-4-3 Beckman Coulter, SLAMF7 Clone 162.1 BioLegend) for 30 min. Unbound antibodies were removed in two washing steps in FACS buffer (1× phosphate-buffered saline (PBS), 10% FBS) and detected via flow cytometry.

### Antibody binding

A total of 5 × 10^5^ MM.1S cells/100 μl cell culture medium were incubated with 500 ng hemibody or BiTE antibody for 1 h at 37 °C. Cells were washed twice with 1× FACS buffer (1× PBS, 10% FBS), pelleted, and resuspended in 100 µl 1× FACS buffer. Bound antibodies were visualized by two labeling steps of 1 µg rabbit polyclonal biotin anti-His Tag antibody (Abcam, Cambridge, UK) for 1 h at 4 °C and 0.5 µl FITC-streptavidin antibody (BioLegend, CA, USA) for 0.5 h at 4 °C via flow cytometry.

### Luciferase-based cytotoxicity assay

A total, 1 × 10^4^ Firefly luciferase-positive MM cells were co-cultured for 20 h with 5 × 10^4^ PBMCs and different constructs in concentrations as indicated at 37 °C, 5% CO_2_.

To analyze the antibody effect on single and dual tumor antigen-positive target cells, 7.5 × 10^3^ firefly luciferase-positive CHO were pre-cultured for 24 h with 7.5 × 10^4^ PBMCs and subsequently with different constructs and concentrations for 20 h at standard cell culture conditions (37 °C, 5% CO_2_).

By the addition of 0.5 mM d-luciferin (Biosynth Inc., USA) for 30 min at 37 °C the antibody-induced tumor cell lysis was measured by the determination of intracellular luciferase activity of living cells. Light emission was quantified with the Infinite M200 PRO ELISA (enzyme-linked immunosorbent assay) Reader (Tecan Group Ltd., Switzerland).

### T cell activation/cytokine ELISA

In total, 1 × 10^4^ Firefly luciferase-positive MM cells were co-cultured with 5 × 10^4^ PBMCs of healthy donors in the presence of antibodies as indicated at standard cell culture conditions (37 °C, 5% CO_2_). Concanavalin A was used as a positive control (Sigma-Aldrich). T cell activation was assessed after 20 h by the secretion of IL-2 and IFN-γ in 50–100 µl supernatant. Commercially available ELISA Kits (IL-2 ELISA Kit, ABIN1446208, antikoerper-online.de, Aachen, Germany, IFN-γ ELISA Kit, hIFNg-EIA-5, MabTag, Friesoythe, Germany) were used according to the manufacturer’s instructions (Infinite M200 PRO ELISA reader, Tecan Group Ltd., Switzerland).

### Nonlinear regression analysis for antibody affinity

The affinity of constructs to CD38 or SLAMF7 protein was analyzed using previously described methods. In total, 15,000 antigen-positive CHO cells/100 µl F12-K medium were plated in 96-well plates and cultured for 24 h at standard cell culture conditions (37 °C, 5% CO_2_). Afterwards, cells were incubated with antibody concentrations as indicated in ELISA buffer (1× PBS, 10% v/v F-12K Nut Mix (1×) culture medium (10% FBS, 1% penicillin/streptomycin, 1% glutamine)) for 2 h at room temperature (RT). Unbound antibodies were removed in two washing steps using 250 µl ELISA buffer and bound antibodies were detected by 50 µl 1:20,000 diluted 6× His Tag HRP antibody (Abcam®) for 1 h at RT, followed by the addition of 50 µl TMB (3,3′,5,5′-tetramethylbenzidine) solution and 50 µl of 1 M H_2_SO_4_ stop solution. Antibody-based absorption was measured at 450 nm by Tecan Spark Reader (Tecan Group Ltd., Switzerland). *K*_D_ values were calculated by a nonlinear regression model using the one-site binding hyperbole and *y* = *B*_max_*x/(*K*_D_ + *x*) (GraphPad Prism 7) (Supplementary Fig. 1c).

### ThermoFluor assay

ThermoFluor assay analysis was performed using SYPRO Orange solution (Sigma-Aldrich, MO, USA), a fluorescent dye that binds to hydrophobic patches exposed as the protein unfolds. A measure of 2.5 µl of 2.5% SYPRO Orange solution was added to 1–5 µg antibody and the thermal stability was detected using the qPCR Cycler CFX Connect™ Real-Time PCR Detection System (Bio-Rad Laboratories Inc., CA, USA) at 472 nm excitation and 570 nm emission. Samples were heated from 25 to 95 °C at 1 °C/min. Data were analyzed using Bio-Rad CFX Manager and GraphPad Prism 7. The thermal transition midpoint (*T*_M_) was calculated by the half-maximal of the antibody unfolding curve (Supplementary Fig. 1d).

### In vivo mouse model

All animal studies received approval from the appropriate authorities (Regierung von Unterfranken, No. RUF-55.2-2531.01-79/11) and comply with all relevant ethical regulations for animal testing and research. For the in vivo model, NSG mice (stock number 5557) were purchased from the Jackson Laboratory (Bar Harbor, ME, USA) and maintained in a certified animal facility (ZEMM, Center for Experimental Molecular Medicine, Würzburg) in accordance with European guidelines.

Six- to 12-week-old female NOD SCID *Il2rg*^−^^/−^ mice were challenged intravenously with 2 × 10^6^ firefly luciferase-positive MM.1S cells. After 14 days and engraftment of tumor cells (day 0 of treatment), 1 × 10^7^ unstimulated PBMCs from a healthy donor were injected intravenously. Mice were treated afterward daily by subcutaneous injection (nuchal fold) of 8 µg hemibodies addressing CD38 and SLAMF7 alone and in combination, SLAMF7 BiTE antibodies, or 1× PBS for 7 days. To monitor the growth of luciferase-positive tumor cells, each mouse received intraperitoneally 220 μl of anesthesia cocktail (Ketavet 8 mg/ml and Xylavet 1.6 mg/ml) and 200 μl luciferin (30 mg/ml) on days 1, 4, and 7 of treatment. Luciferase activity was assessed using an IVIS Lumina XR Real-Time Bioluminescence Imaging System. This analysis was validated in an independent experiment using hemibody and BiTE constructs produced in a eukaryotic expression system and purified/activated with CD3^+^ T cells.

### Statistics and reproducibility

The statistical tests and data analysis methods used in each experiment are given in the figure legends of each figure. In vitro and in vivo assays were not blinded. GraphPad Prism 7 was used for statistical analysis and graphs. All ELISA and cytotoxicity assays were set up in duplicates or triplicates and assayed in several independent experiments. Error bars reflect SEM with *n* = 3 biological triplicates. In all in vitro experiments, “*n*” refers to the number of independent experiments.

### Reporting summary

Further information on research design is available in the Nature Research Reporting Summary linked to this article.

## Supplementary information

Supplementary Information

Description of Additional Supplementary Files

Supplementary Data1

Reporting Summary

## Data Availability

The data that support the findings of this study are available from the corresponding author upon reasonable request. The Source data for graphs and charts in the main figures are provided as Supplementary Data 1.
