# Supplementary material for: Combinatorial targeting of multiple myeloma by complementing T cell engaging antibody fragments
Source: Commun Biol. 2021 Jan 8;4:44. doi: 10.1038/s42003-020-01558-0 (PMC7794243; doi:10.1038/s42003-020-01558-0)
Supplement: Supplementary file 1 — Supplementary Information [file 42003_2020_1558_MOESM1_ESM.pdf]

Combinatorial Targeting of Multiple Myeloma by complementing T cell Engaging Antibody Fragments

Authors: Maria Geis[1], Boris Nowotny[1], Marc-Dominic Bohn[1], Dina Kouhestani[1], Hermann Einsele[1], Thomas Bumm[1], Gernot Stuhler[1*]

Affiliations:

[1]University Clinic Würzburg, Department of Internal Medicine II, Hematology and Oncology, Würzburg, Germany

*Correspondence to: stuhler_g@ukw.de

Supplementary Information:

Supplement Figure 1: Construction and biochemical characteristics of hemibodies.

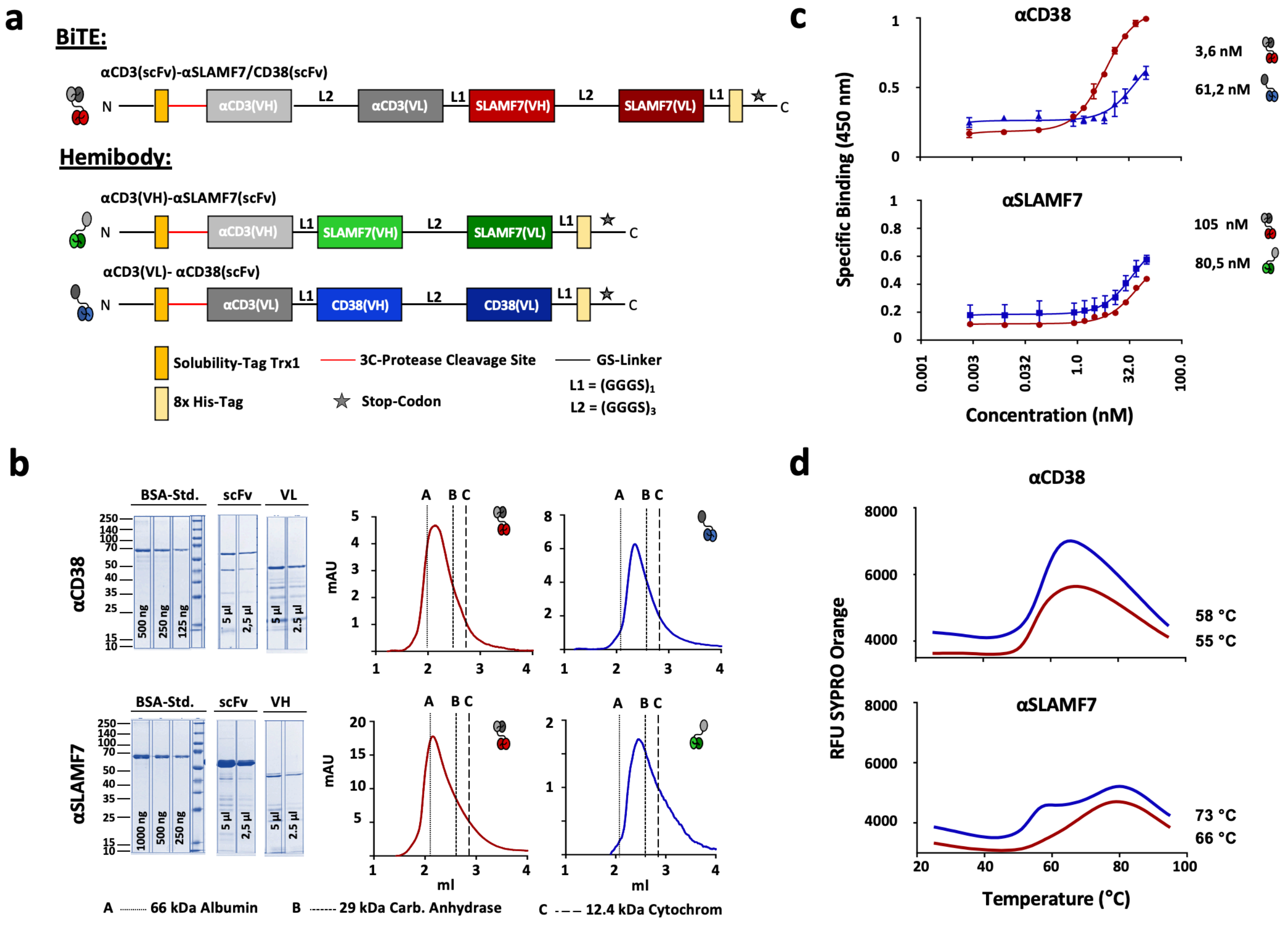

(a) Schematic construction of BiTEs and hemibodies targeting CD38 and SLAMF7. **(b)** Antibodies were isolated by immobilized metal ion affinity chromatography and size-exclusion chromatography. Production yield, purity and monomeric content were analysed by FPLC using 50 ul of size-exclusion eluate and by SDS-PAGE under reducing conditions followed by Coomassie staining in concentrations as indicated (n>3, single experiment is shown). **(c)** Hemibodies were incubated with 10 000 CD38- or SLAMF7-positive CHO cells for 2 h as indicated and the specific antibody binding was detected using ELISA techniques (n=3, single experiment is shown). **(d)** SYPRO Orange based heat stability curve of used antibody constructs from 25-95 °C with a stepwise increase of the temperature of 1 °C/min (n=2-3, single experiment is shown).

Supplement Figure 2: Functional analysis of hemibodies.

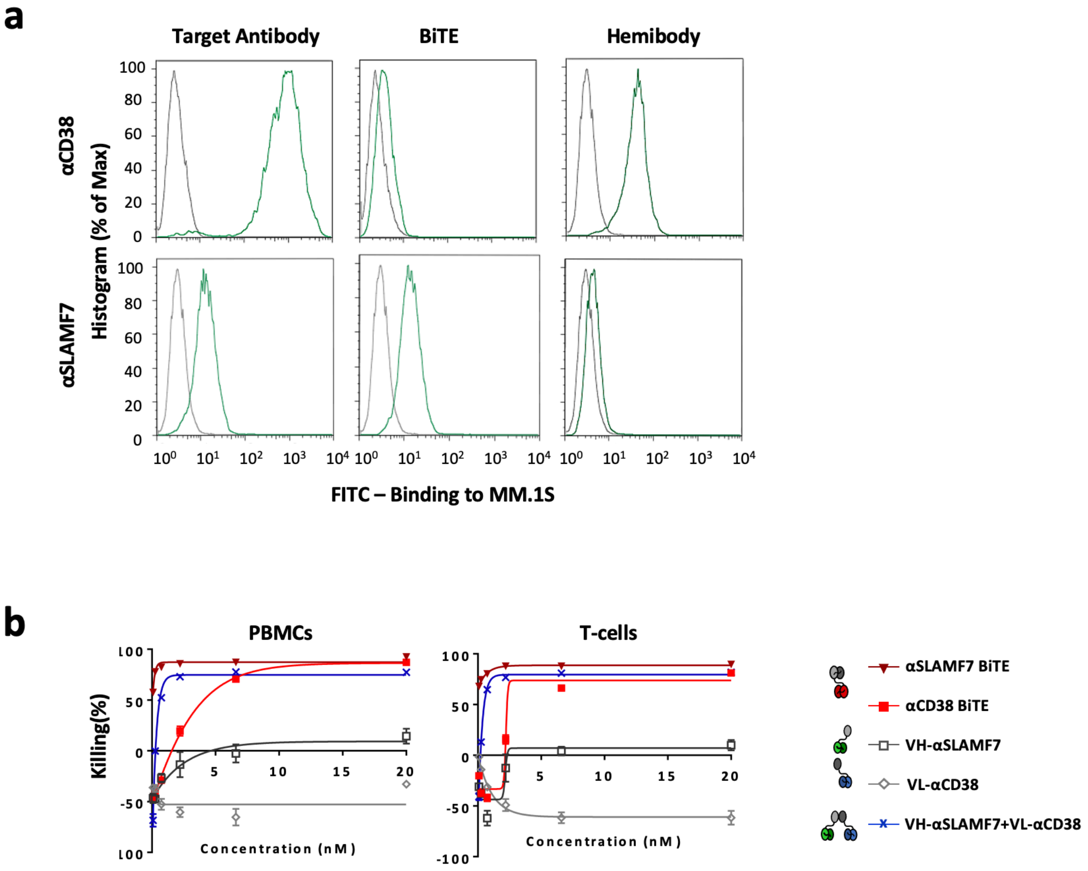

(a) Binding profile of 500 ng hemibody and BiTE constructs to their specific target on MM.1S cells compared to conventional IgG antibodies by flow cytometry (n=3, single experiment is shown). **(b)** Luciferase-positive MM.1S cells were incubated with PBMCs or purified T-cells and different antibody concentrations for 24 h. The T-cell mediated lysis was detected by a luciferase-based cytotoxicity assay (n=3, single experiment is shown).
