# Supplementary material for: Combinatorial targeting of multiple myeloma by complementing T cell engaging antibody fragments
Source: Commun Biol. 2021 Jan 8;4:44. doi: 10.1038/s42003-020-01558-0 (PMC7794243; doi:10.1038/s42003-020-01558-0)
Supplement: Supplementary file 2 — Description of Additional Supplementary Files [file 42003_2020_1558_MOESM2_ESM.pdf]

Description of Additional Supplementary Files

File Name: Supplementary Data 1
Description: Source data for all the main charts and graphs in the main figures.
