# Supplementary material for: Combinatorial targeting of multiple myeloma by complementing T cell engaging antibody fragments
Source: Commun Biol. 2021 Jan 8;4:44. doi: 10.1038/s42003-020-01558-0 (PMC7794243; doi:10.1038/s42003-020-01558-0)
Supplement: Supplementary file 4 — Reporting Summary [file 42003_2020_1558_MOESM4_ESM.pdf]

nature research

Corresponding author(s): Maria Geis, Gernot Stuhler

Last updated by author(s): Nov 12, 2020

Reporting Summary

Nature Research wishes to improve the reproducibility of the work that we publish. This form provides structure for consistency and transparency in reporting. For further information on Nature Research policies, see our Editorial Policies and the Editorial Policy Checklist.

Statistics

For all statistical analyses, confirm that the following items are present in the figure legend, table legend, main text, or Methods section.

n/a	Confirmed	
☐	☒	The exact sample size (n) for each experimental group/condition, given as a discrete number and unit of measurement
☒	☐	A statement on whether measurements were taken from distinct samples or whether the same sample was measured repeatedly
☐	☒	The statistical test(s) used AND whether they are one- or two-sided<br>*Only common tests should be described solely by name; describe more complex techniques in the Methods section.*
☒	☐	A description of all covariates tested
☒	☐	A description of any assumptions or corrections, such as tests of normality and adjustment for multiple comparisons
☐	☒	A full description of the statistical parameters including central tendency (e.g. means) or other basic estimates (e.g. regression coefficient) AND variation (e.g. standard deviation) or associated estimates of uncertainty (e.g. confidence intervals)
☒	☐	For null hypothesis testing, the test statistic (e.g. F, t, r) with confidence intervals, effect sizes, degrees of freedom and P value noted<br>*Give P values as exact values whenever suitable.*
☒	☐	For Bayesian analysis, information on the choice of priors and Markov chain Monte Carlo settings
☒	☐	For hierarchical and complex designs, identification of the appropriate level for tests and full reporting of outcomes
☒	☐	Estimates of effect sizes (e.g. Cohen's d, Pearson's r), indicating how they were calculated

Our web collection on statistics for biologists contains articles on many of the points above.

Software and code

Policy information about availability of computer code

Data collection	Mouse experiments: Living Image Software 4.5.5, In vitro Assays: ImageLa, Tecan SPARKCONTROL Method Editor V2.2 , Unicorn V 6.3, FACSCalibur, Bio-Rad CFX Manager
Data analysis	Mouse experiments: Living Image Software 4.5.5, In vitro Assays: FlowJo V 8.8.6/V10, ImageLab, GraphPad Prism V 7, Unicorn V 6.3, Bio-Rad CFX Manager

For manuscripts utilizing custom algorithms or software that are central to the research but not yet described in published literature, software must be made available to editors and reviewers. We strongly encourage code deposition in a community repository (e.g. GitHub). See the Nature Research guidelines for submitting code & software for further information.

Data

Policy information about availability of data

All manuscripts must include a data availability statement. This statement should provide the following information, where applicable:

- Accession codes, unique identifiers, or web links for publicly available datasets
- A list of figures that have associated raw data
- A description of any restrictions on data availability

The datasets generated during and/or analysed during the current study are available from the corresponding author on reasonable request.

Field-specific reporting

Please select the one below that is the best fit for your research. If you are not sure, read the appropriate sections before making your selection.

[x] Life sciences [ ] Behavioural & social sciences [ ] Ecological, evolutionary & environmental sciences

For a reference copy of the document with all sections, see nature.com/documents/nr-reporting-summary-flat.pdf

Life sciences study design

All studies must disclose on these points even when the disclosure is negative.

Sample size	3 to 4 female mice of same age per group as indicated in respective figures
Data exclusions	-
Replication	-
Randomization	Allocation of samples/animals into experimatal groups was random
Blinding	Blinding was not possible to ensure correct treatment and grouping.

Reporting for specific materials, systems and methods

We require information from authors about some types of materials, experimental systems and methods used in many studies. Here, indicate whether each material, system or method listed is relevant to your study. If you are not sure if a list item applies to your research, read the appropriate section before selecting a response.

Materials & experimental systems

n/a	Involved in the study	
[ ]	[x]	Antibodies
[ ]	[x]	Eukaryotic cell lines
[x]	[ ]	Palaeontology and archaeology
[ ]	[x]	Animals and other organisms
[x]	[ ]	Human research participants
[x]	[ ]	Clinical data
[x]	[ ]	Dual use research of concern

Methods

n/a	Involved in the study	
[x]	[ ]	ChIP-seq
[ ]	[x]	Flow cytometry
[x]	[ ]	MRI-based neuroimaging

Antibodies

Antibodies used	Novus Biologicals/ α-CD38 Clone HB-7 IgG1, κ FITC Cat.No 356610 BioLegend/ α-SLAMF7 (CD319) Clone 162.1 IgG2b, κ FITC Cat.No 331817 BioLegend/ FITC Streptavidin Cat.No 405201 BioLegend/ 6x His tag antibody IgG1, κ Biotin CatNo ab27025 Abcam/ 6x His tag HRP CatNo ab1187 Abcam
Validation	Validation by provider and on myeloma cell lines in different concentrations.

Eukaryotic cell lines

Policy information about cell lines

Cell line source(s)	OPM-2, RPMI-2668, U266 and MM.1S were bought at the German Collection of Microorganisms and Cell Cultures (DSMZ; Braunschweig, Germany)
Authentication	Authentication by provider and light microscope imaging
Mycoplasma contamination	All cell lines were regularly tested negative for mycoplasma contamination.
Commonly misidentified lines (See ICLAC register)	-

Animals and other organisms

Policy information about studies involving animals; ARRIVE guidelines recommended for reporting animal research

Laboratory animals	Immune deficient NOD scid Il2rg-/- (NSG mice), female, age 14 weeks
Wild animals	No wild animals were involved in this study.
Field-collected samples	This study did not involve field-collected samples.
Ethics oversight	ZEMM, center for experimental molecular medicine, Würzburg (experiment number: 2-2544-24)

Note that full information on the approval of the study protocol must also be provided in the manuscript.

Flow Cytometry

Plots

Confirm that:

- [x] The axis labels state the marker and fluorochrome used (e.g. CD4-FITC).
- [x] The axis scales are clearly visible. Include numbers along axes only for bottom left plot of group (a 'group' is an analysis of identical markers).
- [x] All plots are contour plots with outliers or pseudocolor plots.
- [x] A numerical value for number of cells or percentage (with statistics) is provided.

Methodology

Sample preparation	Antibody Binding: 5 x 10^5 MM.1S cells were incubated with 500 ng hemibodie of BiTE antibodies for 1 h at 37 °C. Cells were washed twice with 1x FACS buffer (1x PBS, 10 % FBS), pelleted and resuspended in 100 µl 1x FACS buffer. Bound antibodies were visualized by two labeling steps of 1 µg Rabbit polyclonal Biotin anti-His Tag antibody (Abcam, Cambridge, UK) for 1 h at 4 °C and 0.5 µl FITC Streptavidin antibody (BioLegend, CA, USA) for 0.5 h at 4 °C.
Instrument	BD-FACS Canto-II
Software	FlowJo V 8.8.6/V10
Cell population abundance	1x10°5 of each target cell population were used
Gating strategy	Cells were gated according the light scatter and the target expression (as shown in individual plots)

- [ ] Tick this box to confirm that a figure exemplifying the gating strategy is provided in the Supplementary Information.
